# The value of ACR, European, Korean, and ATA ultrasound risk stratification systems combined with RAS mutations for detecting thyroid carcinoma in cytologically indeterminate and suspicious for malignancy thyroid nodules

**DOI:** 10.1007/s42000-024-00573-8

**Published:** 2024-06-17

**Authors:** Lorenzo Scappaticcio, Nicole Di Martino, Paola Caruso, Pamela Ferrazzano, Federica Zito Marino, Eduardo Clery, Alessandro Cioce, Giovanni Cozzolino, Maria Ida Maiorino, Giovanni Docimo, Pierpaolo Trimboli, Renato Franco, Katherine Esposito, Giuseppe Bellastella

**Affiliations:** 1 Unit of Endocrinology and Metabolic Diseases, AOU University of Campania “Luigi Vanvitelli”, Naples, 80138 Italy; 2 Department of Advanced Medical and Surgical Sciences, University of Campania “Luigi Vanvitelli”, Naples, Italy; 3 Pathology Unit, AOU University of Campania “Luigi Vanvitelli”, Naples, Italy; 4 Unit of Thyroid Surgery, AOU University of Campania “Luigi Vanvitelli”, Naples, Italy; 5 Clinic of Endocrinology and Diabetology, Lugano and Mendrisio Regional Hospital, Ente Ospedaliero Cantonale, Bellinzona, Switzerland; 6 Faculty of Biomedical Sciences, Università della Svizzera Italiana, Lugano, Switzerland

**Keywords:** TIRADS, RAS, Indeterminate thyroid cytology

## Abstract

**Purpose:**

The aim of this study was to evaluate the diagnostic value of four commonly utilized ultrasound (US) RSSs, namely, the American College of Radiology [ACR], European [EU], Korean [K] TI-RADSs and American Thyroid Association [ATA] US-based RSS criteria, in combination with activating point mutations of the RAS genes (NRAS, HRAS, and KRAS) for detection of thyroid carcinoma in cytologically indeterminate and suspicious for malignancy thyroid nodules.

**Methods:**

We retrospectively analyzed cytologically indeterminate and suspicious for malignancy thyroid nodules which underwent US, molecular testing and surgery between September 1, 2018, and December 31, 2023. Receiver operating characteristic (ROC) curves were generated, and the area under the curve (AUC, 95% confidence interval [CI]) was calculated.

**Results:**

A total of 100 cytologically indeterminate and 24 suspicious for malignancy thyroid nodules were analyzed. Compared to the four US-based RSSs alone, the diagnostic value of the four US-based RSSs combined with RAS mutations did not significantly improved (cytologically indeterminate, AUC [95% CI] 0.6 [0.5–0.7] and 0.6 [0.5–0.7], respectively, *p* = 0.70; cytologically suspicious for malignancy, AUC [95% CI] 0.7 [0.5–0.9] and 0.8 [0.6–0.9], respectively, *p* = 0.23).

**Conclusions:**

The diagnostic value of the four main US-based RSSs (ACR, EU, K, and ATA) was not improved in conjunction with the evaluation of RAS mutations for preoperative risk stratification of cytologically indeterminate thyroid nodules.

**Clinical relevance statement:**

In cytologically indeterminate nodules categorized according to US-based RSSs, isolated RAS positivity does not reliably distinguish between benignity and malignancy.

**Supplementary Information:**

The online version contains supplementary material available at 10.1007/s42000-024-00573-8.

## Introduction

Indeterminate cytology represents an important challenge for management of thyroid nodules [1–3]. Neck ultrasound (nUS) is nowadays the first tool to screen thyroid nodules nowadays [1]. US-based risk stratification systems (RSSs) often referred to as thyroid imaging reporting and data systems (TIRADSs) exhibit high diagnostic performance in papillary thyroid cancer (PTC) and its classical variant [4, 5]. In fact, however, the diagnostic performance of TIRADs for the above cancers, which are often labeled as being of indeterminate cytology, is deemed to be moderate because of their overall non-high suspicion US features [5–9]. Fine-needle cytology (FNC) remains the gold standard for nonsurgical evaluation of thyroid nodules [1, 10]. Nonetheless, up to 25–30% of FNCs render indeterminate cytology [1]. Many of these nodules ultimately lead to a diagnostic surgery, which finds 70% of them to be histologically benign and unnecessarily exposed to potential surgical risks and to lifelong need for thyroid hormone replacement therapy [9, 11].

The role of molecular testing in thyroid nodules, which is currently an evolving procedure, can be used in combination with cytology and ultrasound characteristics as an adjuvant test [1]. It is recommended that the molecular markers are recommended to be used in cases with indeterminate and suspicious for malignancy cytologic diagnoses to guide management [1, 2, 12]. Molecular testing can either be a rule-in test with a high positive predictive value and specificity or a rule-out test with a high negative predictive value and sensitivity [13]. Large molecular panels (i.e., Afirma GSC, ThyroSeq v3, and ThyGeNEXT/ThyraMIR) are very rarely used in Europe due to their high price and reimbursement issues; meanwhile, the panels used in clinical practice target only a few genes, including RAS, BRAF, RET/PTC, and PAX8/PPARɣ [3, 14, 15]. Different types of molecular alterations can be detected in thyroid nodules, certain of them (i.e., BRAF V600E) being more frequently associated with malignancy than others [3, 16–18]. The RAS genes family (NRAS, HRAS, and KRAS) mutations represent the majority (about 70%) of all mutated cases, especially in indeterminate cytology, and they can be seen in histologically benign and malignant thyroid nodules [9, 14]. In fact, the risk of malignancy associated with RAS mutations has been reported to be between 23 and 76% [9, 19, 20].

The management of nodules carrying RAS-like mutations is still under debate [19, 21] and limited data exist about the impact of RAS mutations on ultrasound risk stratification [9, 19, 22, 23]. The aim of this study was to evaluate the diagnostic value of four commonly utilized ultrasound RSSs (the American College of Radiology [ACR], European [EU], Korean [K] TI-RADSs and American Thyroid Association [ATA] US-based RSS criteria) [1, 24–26] in combination with activating point mutations of the RAS gene (NRAS, HRAS, and KRAS) for detecting thyroid carcinoma in cytologically indeterminate and suspicious for malignancy thyroid nodules at the authors’ institution.

## Methods

### Study design and patients

The Standards for Reporting Diagnostic Accuracy (STARD) statement was followed [27]. In our Academic referral center, a retrospective retrieval of consecutive FNC results from adult patients with cytologically indeterminate and suspicious for malignancy thyroid nodules which underwent molecular testing between September 1, 2018, and December 31, 2023 was carried out.

Nodules were included as follows when: (a) they had indeterminate (i.e., low-risk [TIR3A] and high-risk [TIR3B]) or suspicious for malignancy (TIR4) cytology; (b) cytological samples were available and adequate for molecular DNA analysis; (c) the matched histology after surgery was available; and (d) the ACR, European, Korean and ATA ultrasound risk stratification systems were separately applied to categorize each nodule from at least two clear B-Mode US images (i.e., transverse, and longitudinal images). Nodules were excluded as follows: when (a) they had malignant or inadequate or benign cytology; (b) they had other mutations rather than RAS mutations; (c) they were associated with specific malignant histology (i.e., medullary thyroid carcinoma; poorly differentiated and anaplastic thyroid carcinoma; or thyroid lymphoma); (d) there was unavailability of well-preserved and adequate cytological samples; and (e) surgery was not performed at our center.

In our center, FNC was performed according to ultrasound risk stratification systems [1, 24–26] and thyroid scintigraphy [i.e., FNC was not performed in autonomous functioning thyroid nodules (AFTNs)] and/or the clinician’s and patient’s preference [3]. Molecular testing was added to the first (routine test) or second FNC according to clinical judgment in cytologically indeterminate, suspicious for malignancy, and malignant thyroid nodules. Surgery was recommended according to ultrasound, cytological and molecular results, clinician’s and patient’s preference, and underlying symptomatic benign multinodular goiter or Graves’ disease [3].

### Thyroid ultrasonography

At our center US images were obtained using an ultrasound device (MyLab™Six, Esaote) with a 7–14 MHz wide band linear transducer. The color gain was adjusted so that artifacts were prevented. When reviewing the US images on digital format, two endocrinologists (G.B. and L.S.) assessed the thyroid nodules by using the criteria of the four ultrasound RSSs (ACR-, EU-, K-TI-RADSs and ATA US-based RSS criteria) while being unaware of nodule’s cytopathology and histopathology and of laboratory and imaging results. In event of disagreement on US categorization, consensus was reached with the help of a third senior reviewer (P.T.) (also unaware of pathology or any other patient data) was reached.

### Thyroid nodule pathology

Cytologic diagnoses were reported according to the five subcategories of the revised Italian Consensus for the Classification and Reporting of Thyroid Cytology (ICCRTC) [12]. All the available slides from each case were reviewed by two thyroid cytopathologists (R.F. and E.C.). When reviewing cytology specimens, pathologists were unaware of demographics and clinical data, including US features of thyroid nodules. The two pathologists (R.F. and E.C.) were unaware of histopathologic diagnoses. The final pathology report (i.e., histology of the thyroid nodule after surgery) was made according to the 2022 WHO Classification of Thyroid Neoplasms, this also being applied to cases before 2022 which were accordingly reclassified [28]. Non-invasive follicular thyroid neoplasms with papillary-like nuclear feature (NIFTPs) were considered as malignant cases, although it is known that these are associated with low-risk aggressiveness [28].

### Molecular analysis

RAS mutations were assessed as part of a panel of molecular genetic tests including RAS, BRAF, RET/PTC, and PAX8/ PPARɣ. Thyroid FNCs were processed to extract DNA and RNA simultaneously using the AllPrep DNA/RNA Kit (Qiagen, Hilden, Germany), following the manufacturer’s instructions. The nucleic acids were eluted in 30 µL of nuclease-free water. Detection of HRAS, NRAS, KRAS and BRAF somatic mutations in the genomic DNA isolated from thyroid cytology specimens was performed via Real-Time PCR using the EasyPGX ready THYROID (Diatech Pharmacogenetics). The assays include amplification of the endogenous control gene which enables verification the quality and quantity of the nucleic acids, the amplification procedures, and the possible presence of inhibitors.

FNCs were defined as RAS positive (+) if harboring activating mutations in one of RAS genes (NRAS or HRAS or KRAS), whereas WILD type if no mutations were detected according to our assay.

### Statistical analysis

Quantitative variables are presented as median (interquartile range [IQR], 1st and the 3rd quartiles), while qualitative variables are presented as absolute and relative (%) frequencies. To calculate the p-value for trend (t^2^), the Cochran-Armitage trend test was used. Diagnostic value (sensitivity [SE], specificity [SPEC], positive predictive value [PPV], negative predictive value [NPV], accuracy) were calculated considering cyto-histological correlations as reference. For calculation of the diagnostic value of the four TIRADSs and the RAS testing high risk (category 5) categories and the presence of a RAS mutation (+) were considered as positive cases, respectively. Receiver operating characteristic (ROC) curves were generated, and the area under the curve (AUC, 95% confidence interval [CI]) was calculated to facilitate comparative analysis. Benign and suspicious call rates (BCR and SCR) were the molecular marker test outcome that were assigned after the sonographic categorization. Statistical significance was established at a significance level of *p* < 0.05. Med Calc v22.017–64 bit (MedCalc Software Ltd), Sigma Stat (version 3.5) and IBM SPSS (version 29) were utilized.

## Results

### Whole cohort characteristics

**Fig. 1:**
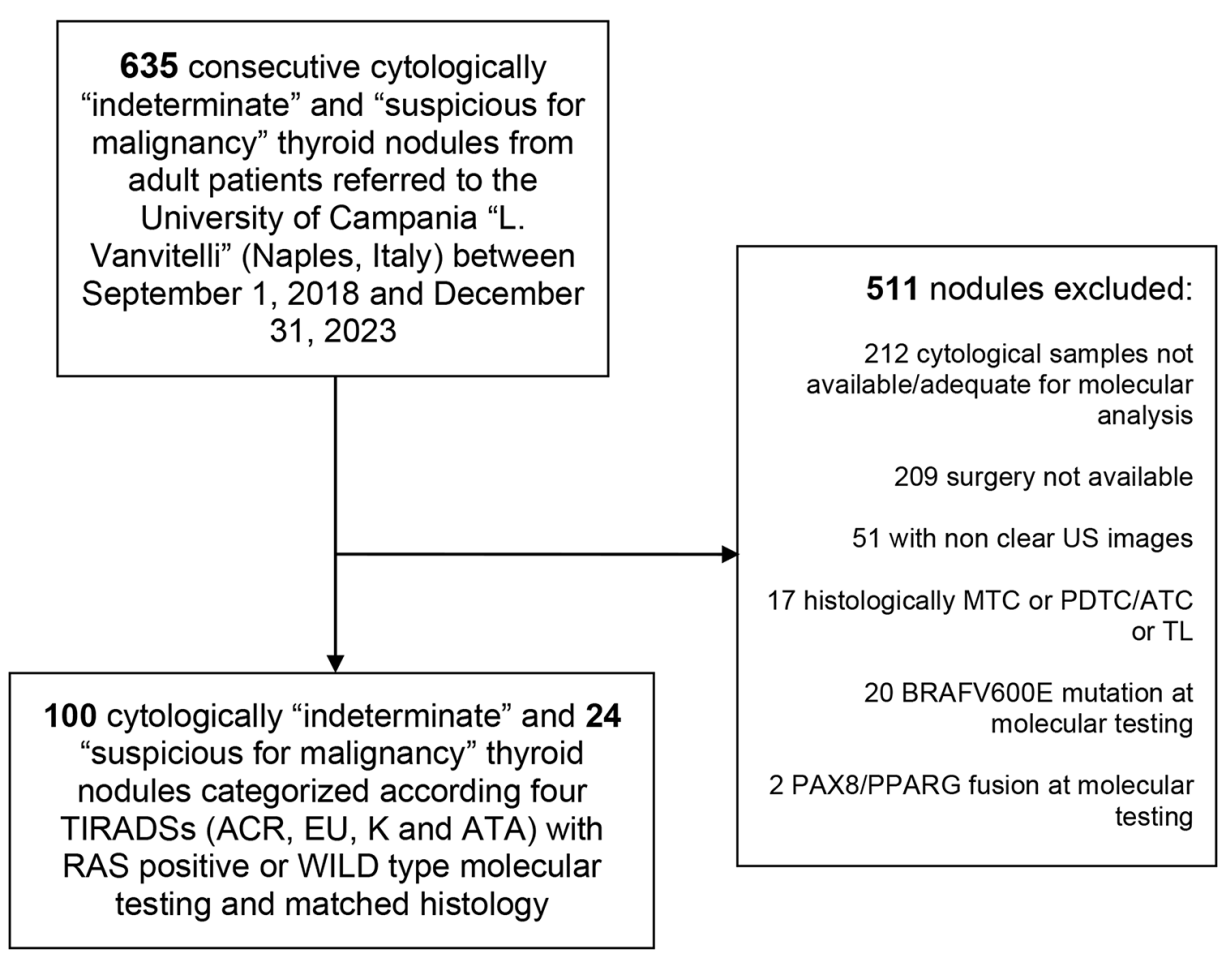
Flowchart of patients’ selection. US, ultrasound; TIRADSs, Thyroid Imaging Reporting and Data Systems; ACR, American College of Radiology; EU, European; K, Korean; ATA, American Thyroid Association; MTC, medullary thyroid carcinoma; PDTC/ATC, poorly differentiated and anaplastic thyroid carcinoma; TL, thyroid lymphoma

We finally reviewed 100 cytologically indeterminate (TIR3A n: 55 and TIR3B n: 45) and 24 suspicious for malignancy(TIR4) thyroid nodules from 124 patients in compliance with four TIRADSs (ACR, EU, K and ATA US-based RSS) (Fig. 1). Fifty-eight out of 124 nodules (46.7%) were RAS positive. Table 1 summarizes the main characteristics of the whole cohort (n: 124).

**Table 1:** Main characteristics of the whole cohort (n: 124)

Characteristics							
Age at diagnosis, years (IQR)	53 (41-60.5)						
Females/Males, n (ratio)	86/38 = 2.3						
Maximal dimension, mm (IQR)	20 (13-28.5)						
Location, n (%)							
• Right lobe	62 (50)						
• Left lobe	54 (43.6)						
• Isthmus	8 (6.4)						
Histology, n (%)							
• Benign	68 (54.8)						
• Malignant	56 (45.2)						
Cytology, n (%)		**RAS +** ^d^, n (%)		**Malignancy rate**, n (%)			
• TIR3A^a^	55 (44.4)	30 (24.2)		20 (36.4)			
• TIR3B^b^	45 (36.3)	18 (14.5)		14 (31.1)			
• TIR4^c^	24 (19.3)	10 (8.0)		22 (91.7)			
		Tot 58 (46.7)		*p* < 0.001, *p t*^*2*^ 0.0003			
• TIR3	100 (80.7)	48 (38.7)		34 (34.0)			
ACR TI-RADS,							
• 5, n (%)	16 (12.9)	10 (8.0)		87.5			
• 4, n (%)	54 (43.6)	26 (21.0)		51.9			
• 3, n (%)	50 (40.3)	18 (14.5)		20			
• 2, n (%)	4 (3.2)	4 (3.2)		100			
				*p* < 0.001, *p t*^*2*^ 0.003			
EU-TIRADS,							
• 5, n (%)	16 (12.9)	10 (8.0)		87.5			
• 4, n (%)	49 (39.5)	24 (19.4)		46.9			
• 3, n (%)	55 (44.4)	20 (16.1)		27.3			
• 2, n (%)	4 (3.2)	4 (3.2)		100			
				*p* < 0.001, *p t*^*2*^ 0.003			
K-TIRADS,							
• 5, n (%)	16 (12.9)	10 (8.0)		87.5			
• 4, n (%)	49 (39.5)	24 (19.4)		46.9			
• 3, n (%)	55 (44.4)	20 (16.1)		27.3			
• 2, n (%)	4 (3.2)	4 (3.2)		100			
				*p* < 0.001, *p t*^*2*^ 0.003			
ATA US-based RSS,							
• High, n (%)	16 (12.9)	10 (8.0)		87.5			
• Intermediate, n (%)	49 (39.5)	24 (19.4)		46.9			
• Low, n (%)	55 (44.4)	20 (16.1)		27.3			
• Very low, n (%)	4 (3.2)	4 (3.2)		100			
				*p* < 0.001, *p t*^*2*^ 0.003			

*IQR*, interquartile range (1st and the 3rd quartiles); n, number (count); mm, millimeter; tot, total; *p*, p value; *pt*^2^, p for trend^2^; *ACR, EU, K, TI-RADS*, American College of Radiology, European, Korean, Thyroid Imaging Reporting and Data System; ATA US-based RSS, American Thyroid Association ultrasound-based risk stratification system

Quantitative variables are presented as median (IQR)

^a^TIR3A, low-risk indeterminate according to the Italian Consensus for the Classification and Reporting of Thyroid Cytology [12]

^b^TIR3B, high-risk indeterminate according to the Italian Consensus for the Classification and Reporting of Thyroid Cytology [12]

^c^TIR4, suspicious for malignancy according to the Italian Consensus for the Classification and Reporting of Thyroid Cytology [12]

^d^RAS positive (+) were mutated cases with isolated RAS (NRAS or HRAS or KRAS) mutation

### Malignancy rates (MRs) according to cytology, TIRADSs and RAS gene mutations

Table 2 shows the MRs of the whole cohort according to both cytology and TIRADSs.

**Table 2:** Malignancy rates of the whole cohort (n: 124) according to cytology and TIRADSs

	Malignancy rate, *n* (%)			
ACR TI-RADS	TIR4	TIR3A	TIR3B	TIR3 (TIR3A + TIR3B)
2	0 (0)	0 (0)	4 (100)	4 (100)
3	2 (66.7)	6 (20.0)	2 (11.8)	8 (17.0)
4	11 (91.7)	12 (54.5)	5 (25.0)	17 (40.5)
5	9 (100)	2 (66.7)	3 (75.0)	5 (71.4)
**EU-TIRADS**				
2	0 (0)	0 (0)	4 (100)	4 (100)
3	5 (83.3)	8 (25.0)	2 (11.8)	10 (20.4)
4	8 (88.9)	10 (50.0)	5 (25.0)	15 (37.5)
5	9 (100)	2 (66.7)	3 (75.0)	5 (71.4)
**K-TIRADS**				
2	0 (0)	0 (0)	4 (100)	4 (100)
3	5 (83.3)	8 (25.0)	2 (11.8)	10 (20.4)
4	8 (88.9)	10 (50.0)	5 (25.0)	15 (37.5)
5	9 (100)	2 (66.7)	3 (75.0)	5 (71.4)
**ATA US-based RSS**				
Very low	0 (0)	0 (0)	4 (100)	4 (100)
Low	5 (83.3)	8 (25.0)	2 (11.8)	10 (20.4)
Intermediate	8 (88.9)	10 (50.0)	5 (25.0)	15 (37.5)
High	9 (100)	2 (66.7)	3 (75.0)	5 (71.4)

n, number (count); *ACR, EU, K, TI-RADS*, American College of Radiology, European, Korean, Thyroid Imaging Reporting and Data System; ATA US-based RSS, American Thyroid Association ultrasound-based risk stratification system

^a^TIR3A, low-risk indeterminate according to the Italian Consensus for the Classification and Reporting of Thyroid Cytology [12]

^b^TIR3B, high-risk indeterminate according to the Italian Consensus for the Classification and Reporting of Thyroid Cytology [12]

^c^TIR4, suspicious for malignancy according to the Italian Consensus for the Classification and Reporting of Thyroid Cytology [12]

Table 3 shows the MRs of the RAS + cohort (30/58, 51.7%) according to both cytology and RAS gene (NRAS or HRAS or KRAS) mutation.

**Table 3:** Malignancy rates of the RAS + cohort (n: 58) according to cytology and RAS gene (NRAS or HRAS or KRAS) mutation

	Malignancy rate, *n* (%)						
	TIR4	TIR3A	TIR3B	TIR3 (TIR3A + TIR3B)	Overall		
**NRAS** (n 39)	6 (100)	9 (39.1)	7 (70.0)	16 (48.5)	22 (56.4)		
**HRAS** (n 15)	2 (100)	4 (66.7)	0 (0)	4 (30.8)	6 (40.0)		
**KRAS** (n 4)	2 (100)	0 (0)	0 (0)	0 (0)	2 (50.0)		
Total (n 58)					30 (51.7)		

RAS positive (+) were mutated cases with isolated RAS (NRAS or HRAS or KRAS) mutation

n, number (count)

TIR3A, low-risk indeterminate according to the Italian Consensus for the Classification and Reporting of Thyroid Cytology [12]

TIR3B, high-risk indeterminate according to the Italian Consensus for the Classification and Reporting of Thyroid Cytology [12]

TIR4, suspicious for malignancy according to the Italian Consensus for the Classification and Reporting of Thyroid Cytology [12]

### Histology of the whole cohort according to cytology and molecular testing

As reported in Supplementary Table 1, the 56 malignant cases were as follows: 27 papillary thyroid carcinomas (PTC) (16 RAS +, 11 WILD type), 14 NIFTPs (8 RAS +, 6 WILD type), 13 follicular thyroid carcinomas (FTCs) (6 RAS +, 7 WILD type), 2 well-differentiated tumors of uncertain malignant potential (WDT-UMPs) (both WILD type).

### Diagnostic value of RAS mutations

Table 4 reports the diagnostic value of RAS mutations.

**Table 4:** Diagnostic value of RAS mutation (NRAS or HRAS or KRAS) in cytologically indeterminate and suspicious for malignancy thyroid nodules

	SE	SPEC	NPV	PPV	Accuracy	AUC (95% CI)
TIR4	45.4	100	14.3	100	50.0	0.7 (0.5–0.9)
TIR3A	65.0	51.4	72.0	43.3	56.4	0.6 (0.4–0.7)
TIR3B	50.0	64.5	74.1	38.9	60.0	0.6 (0.4–0.7)
TIR3	58.8	57.6	73.1	41.7	58.0	0.6 (0.4–0.7)

SE, sensitivity; SPEC, specificity; PPV, positive predictive value; NPV, negative predictive value; AUC, area under the curve

TIR3A, low-risk indeterminate according to the Italian Consensus for the Classification and Reporting of Thyroid Cytology [12]

TIR3B, high-risk indeterminate according to the Italian Consensus for the Classification and Reporting of Thyroid Cytology [12]

TIR4, suspicious for malignancy according to the Italian Consensus for the Classification and Reporting of Thyroid Cytology [12]

TIR3 includes both TIR3A and TIR3B cases

### Diagnostic value of the four TIRADSs

Table 5 reports the diagnostic value of the four TIRADSs.

**Table 5:** Diagnostic value of ACR TI-RADS, EU-TIRADS, K-TIRADS, ATA US-based RSS and their combination with RAS mutation (NRAS or HRAS or KRAS) in cytologically indeterminate and suspicious for malignancy thyroid nodules

		SE	SPEC	NPV	PPV	Accuracy	AUC (95% CI)	*p*-value
TIR4	ACR	40.9	100	13.3	100	45.8	0.7 (0.5–0.9)	0.23
	ACR + RAS	59.1	100	18.2	100	62.5	0.8 (0.6–0.9)	
	EU	40.9	100	13.3	100	45.8	0.7 (0.5–0.9)	0.23
	EU + RAS	59.1	100	18.2	100	62.5	0.8 (0.6–0.9)	
	K	40.9	100	13.3	100	45.8	0.7 (0.5–0.9)	0.23
	K + RAS	59.1	100	18.2	100	62.5	0.8 (0.6–0.9)	
	ATA	40.9	100	13.3	100	45.8	0.7 (0.5–0.9)	0.23
	ATA + RAS	59.1	100	18.2	100	62.5	0.8 (0.6–0.9)	
TIR3A	ACR	10.0	97.1	65.4	66.7	65.5	0.5 (0.4–0.7)	0.69
	ACR + RAS	65.0	48.6	70.8	41.9	54.5	0.6 (0.4–0.7)	
	EU	10.0	97.1	65.4	66.7	65.5	0.5 (0.4–0.7)	0.69
	EU + RAS	65.0	48.6	70.8	41.9	54.5	0.6 (0.4–0.7)	
	K	10.0	97.1	65.4	66.7	65.5	0.5 (0.4–0.7)	0.69
	K + RAS	65.0	48.6	70.8	41.9	54.5	0.6 (0.4–0.7)	
	ATA	10.0	97.1	65.4	66.7	65.5	0.5 (0.4–0.7)	0.69
	ATA + RAS	65.0	48.6	70.8	41.9	54.5	0.6 (0.4–0.7)	
TIR3B	ACR	21.4	96.8	73.2	75.0	73.3	0.6 (0.4–0.7)	0.99
	ACR + RAS	57.1	61.3	76.0	40.0	60.0	0.6 (0.4–0.7)	
	EU	21.4	96.8	73.2	75.0	73.3	0.6 (0.4–0.7)	0.99
	EU + RAS	57.1	61.3	76.0	40.0	60.0	0.6 (0.4–0.7)	
	K	21.4	96.8	73.2	75.0	73.3	0.6 (0.4–0.7)	0.99
	K + RAS	57.1	61.3	76.0	40.0	60.0	0.6 (0.4–0.7)	
	ATA	21.4	96.8	73.2	75.0	73.3	0.6 (0.4–0.7)	0.99
	ATA + RAS	57.1	61.3	76.0	40.0	60.0	0.6 (0.4–0.7)	
TIR3	ACR	14.7	97.0	68.8	71.4	69.0	0.6 (0.5–0.7)	0.70
	ACR + RAS	61.8	54.5	73.5	41.2	57.0	0.6 (0.5–0.7)	
	EU	14.7	97.0	68.8	71.4	69.0	0.6 (0.5–0.7)	0.70
	EU + RAS	61.8	54.5	73.5	41.2	57.0	0.6 (0.5–0.7)	
	K	14.7	97.0	68.8	71.4	69.0	0.6 (0.5–0.7)	0.70
	K + RAS	61.8	54.5	73.5	41.2	57.0	0.6 (0.5–0.7)	
	ATA	14.7	97.0	68.8	71.4	69.0	0.6 (0.5–0.7)	0.70
	ATA + RAS	61.8	54.5	73.5	41.2	57.0	0.6 (0.5–0.7)	

SE, sensitivity; SPEC, specificity; PPV, positive predictive value; NPV, negative predictive value; AUC, area under the curve; ACR, EU, K, TI-RADS, American College of Radiology, European, Korean, Thyroid Imaging Reporting and Data System; ATA US-based RSS, American Thyroid Association ultrasound-based risk stratification system

TIR3A, low-risk indeterminate according to the Italian Consensus for the Classification and Reporting of Thyroid Cytology [12]

TIR3B, high-risk indeterminate according to the Italian Consensus for the Classification and Reporting of Thyroid Cytology [12]

TIR4, suspicious for malignancy according to the Italian Consensus for the Classification and Reporting of Thyroid Cytology [12]

TIR3 includes both TIR3A and TIR3B cases

### Diagnostic value of the four TIRADSs combined with RAS mutations

**Fig. 2:**
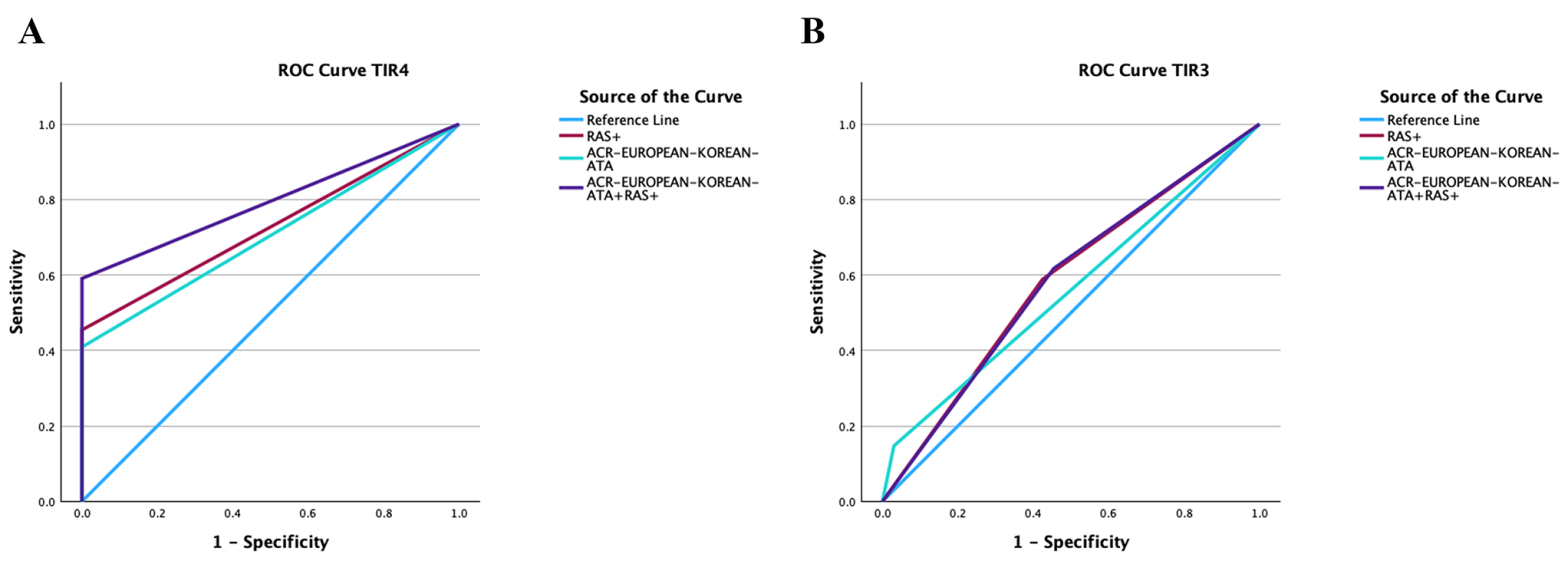
Receiver operating characteristic (ROC) curves for ACR, European, Korean and ATA ultrasound risk stratification systems combined with RAS testing in cytologically suspicious for malignancy (TIR4) (**A**) and indeterminate (TIR3) (**B**) thyroid nodules

In TIR4 nodules, for each TIRADS the AUC (95% CI) was not different from when it was combined with RAS testing (0.7 [0.5–0.9] and 0.8 [0.6–0.9], respectively, *p* = 0.23). In TIR3 nodules, for each TIRADS the AUC (95% CI) was not different from when was combined with RAS testing (0.6 [0.5–0.7] and 0.6 [0.5–0.7], respectively, *p* = 0.70) (Table 5; Fig. 2).

**Fig. 3:**
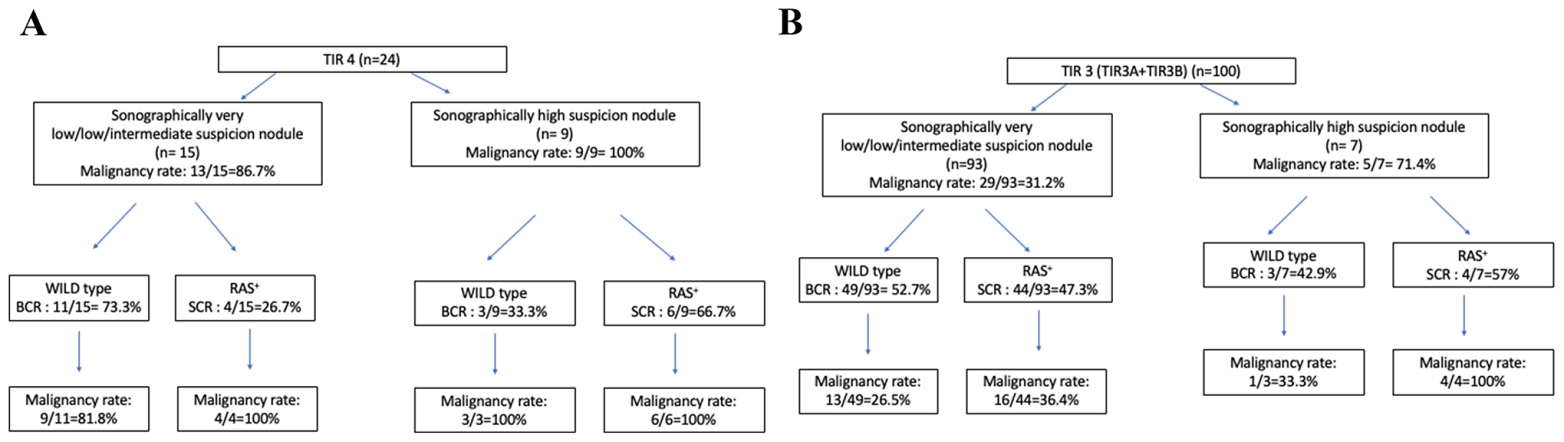
Malignancy rates stratified by sonographic risk categorizations according to ACR, European, Korean and ATA ultrasound risk stratification systems alone and by sonographic risk categorizations in conjunction with RAS test results (**A** for TIR4 nodules and **B** for TIR3 nodules). In the first branch, malignancy rates were reported for sonographic risk categories alone (sonographically very low/low/intermediate suspicion nodules and sonographically high suspicion nodules) before RAS test results (pretest malignancy rates). Nodules from each sonographic category were then assigned a RAS marker test outcome (BCRs and SCRs). New malignancy rates were reported for each sonographic risk category after receiving RAS test results (post-test malignancy rates). BCR, Benign call rates; SCR, suspicious call rates

In TIR4 nodules three TIRADSs (ACR, EU, K, ATA) high suspicion nodules received a WILD type testing result (BCR = 33.3%) and they were found to be malignant on histopathology (Fig. 3A). In TIR4 nodules 11 TIRADSs (ACR, EU, K, ATA) very low/low/intermediate suspicion nodules received a WILD type testing result (BCR = 73.3%) and nine were found to be malignant on histopathology so that the post-test malignancy rate became 81.8% (Fig. 3A).

In TIR3 nodules three TIRADSs (ACR, EU, K, ATA) high suspicion nodules received a WILD type testing result (BCR = 42.9%) and one was found to be malignant on histopathology so that post-test malignancy rate became 33.3% (Fig. 3B). In TIR3 nodules 49 TIRADSs (ACR, EU, K, ATA) very low/low/intermediate suspicion nodules received a WILD type testing result (BCR = 52.7%) and 13 were found to be malignant on histopathology so that the post-test malignancy rate became 26.5% (Fig. 3B).

## Discussion

RAS mutation was present in almost half of indeterminate nodules, a finding that confirms the existing evidence [9, 14, 29]. Moreover, as already reported in several studies [7, 24, 30], the nodules were mainly distributed in the low and indeterminate categories of TIRADSs, and only about one out of 10 nodules was sonographically high suspicion. In agreement with the expected figures in TIRADSs [1, 24–26], MRs were higher in sonographically high suspicion nodules compared to sonographically low and intermediate suspicion nodules. However, in TIR3 nodules we found 100% of MR in sonographically very low suspicion nodules. These were four high risk (TIR3B) indeterminate nodules that were categorized as very low suspicion nodules according to the four TIRADSs but with malignant histology (two minimally invasive FTC and two tall cell subtype of PTC). This was moreover not surprising, since we know that TIRADSs perform suboptimally in FTC and the non-classical subtype of PTC [5, 6]. NRAS was the most common RAS mutation as it was detected in about two out of three RAS + nodules, this being in line with the current literature [14, 15, 20]. However, we did not find differences in MRs across the three isoforms of RAS, contrary to what other studies have reported (i.e., decreasing rates of malignancy from HRAS to NRAS to KRAS) [9, 31, 32]. As regards the histology of the 56 malignant cases, almost 50% were PTCs, the other 50% were equally distributed in NIFTPs and FTCs, and other 2 cases were WDT-UMPs. Specifically, among the 30 RAS + malignant cases (the remaining 28 RAS + cases were benign) there were eight classic PTCs, six fv PTCs, and eight NIFTPs, so that more than seven out of ten cases were potentially not aggressive thyroid cancers. The latter finding was already reported in other studies, according to which malignancy of RAS mutation alone is most likely associated with limited aggressiveness of thyroid cancer [31, 33]. In no case we did detect more than one mutation beyond that in RAS genes, and we included only BRAFV600E mutation–negative nodules.

In the present series, the accuracy of RAS testing was moderate irrespective of cytology (58% in cytologically indeterminate and 50% in suspicious for malignancy thyroid nodules). We found that RAS mutations had both specificity and PPV of 100% in cytologically suspicious for malignancy thyroid nodules, where MR was 91.7%. However, low values of SE (45.4%) and NPV (14.3%) were observed among this subgroup, so that also in cytologically suspicious for malignancy nodules RAS testing could not represent a reliable rule-out test. In cytologically indeterminate subgroup, where MR was 34%, SE and PPV of RAS testing were low (below 60% and 45%, respectively), affecting its accuracy as a rule-in and rule-out test. Similarly, low values of both SE and PPV were recently reported by Wu et al. [23], who explored the diagnostic value of KRAS mutation in cytologically indeterminate nodules. We therefore confirmed that RAS mutations were not specific for malignancy.

As concerns TIRADSs, at the high risk (category 5) ultrasound category threshold, we found overlapping diagnostic value of four TIRADSs (ACR, EU, K and ATA US-based RSS) alone and in combination with RAS testing, and irrespective of cytology. TIRADSs alone showed moderate accuracy (69.0% in cytologically indeterminate and 45.8% in suspicious for malignancy thyroid nodules) since they were unsatisfactory as “rule-out test”. In other words, although we found a relatively high SPEC and PPV for the preoperative ultrasonographic identification of nodules with suspicious features, most of the histologically follicular cancers evaluated in this series were missed by ultrasound. Our findings showed that while suspicious ultrasonographic features are useful when present, most RAS-positive cancers lack suspicious features of any type. These findings were in line with those of Wang et al. [30].

Moreover, when we explored the value of the four TIRADSs in combination with RAS testing we obtained a non-significant improvement of accuracy in cytologically suspicious for malignancy thyroid nodules and a non-significant worsening of accuracy in cytologically indeterminate nodules (Table 5). Specifically, in cytologically indeterminate nodule, although being an absolute amelioration of SE, we found a relevant decrease in SPEC and PPV.

As illustrated in Fig. 3, our final aim was to show how TIRADSs and RAS testing could integrate in a clinical scenario. Specifically, in cytologically suspicious for malignancy thyroid nodules, when dealing with sonographically high suspicion nodules RAS mutation could not improve malignancy rates. This was due to the high specificity for malignancy of TIRADSs in this context and the possibility to have WILD type testing result being malignant on histology (three cases in our study). Conversely, when facing sonographically non-high suspicion nodules RAS testing improperly would avoid surgery in 9 out of eleven WILD type nodules, while correctly would indicate surgery in 4 out of 4 RAS + cases. Therefore, this altogether meant that, in cytologically suspicious for malignancy thyroid nodules, RAS testing would have correctly diagnosed malignancy in only one case more than the four TIRADSs. In cytologically indeterminate thyroid nodules, when facing sonographically high suspicion nodules RAS testing correctly diagnosed four malignancies while improperly would avoid surgery in three cases. Furthermore, when facing sonographically non-high suspicion nodules if on one side RAS testing correctly indicated surgery in 16 cases and correctly avoided surgery in 36 cases, on the other side it improperly indicated surgery in 28 cases and improperly avoided surgery in 13 cases. Therefore, this altogether meant that, in cytologically indeterminate thyroid nodules, RAS testing would have correctly diagnosed malignancy in 15 cases more than the four TIRADSs but would have improperly indicated surgery in 26 cases more than the four TIRADSs. All this meant that RAS mutations and US-based RSSs, taken singly, are limited at differentiating malignant from benign indeterminate nodules, as well as their combination. The major limitation of both suspicious sonographic features and/or mutational markers was their relatively low occurrence in malignant indeterminate nodules [22].

There are limitations of our study that warrant some caution. First, there was an unavoidable selection bias because the data were retrospectively evaluated, and surgery and molecular testing were not always available. Second, the study has a single-center experience that cannot account for geographic variations in incidences of thyroid cancer or thyroid cancer subtypes. Third, our findings only referred to isolated RAS mutations, failing to explore the diagnostic value of RAS mutations with other molecular alterations [19, 21]. Fourth, this series was limited to nodules with operative treatment, which may have contributed to referral bias. Fifth, our results derived from the ultrasound data could be different in other centers also because of the moderate interobserver variability [34]. However, the use of US-based RSSs is deemed to significantly improve interobserver agreement in assessing thyroid nodules [35]. Sixth, this study relied on static image analysis, which could introduce a certain degree of bias during the classification process.

Our study’s strengths are multiple. First, all the histologic outcomes were after the advent of NIFTP [36], properly reporting the final diagnosis and avoiding false diagnoses of follicular variant of PTC [33]. Second, our findings were representative of the four main US-based RSSs for the first time, alone and in combination with RAS mutations, since we tested two pattern-based TIRADSs (ETA and ATA) and two score-based TIRADSs (ACR and KOREAN), and we found overlapping results among these four RSSs. Third, our protocol was designed to also explore diagnostic value of cytologically suspicious for malignancy nodules, since future investigations into clinical utility of RAS mutations across other cytological categories are warranted to strengthen the evidence of ATA guideline recommendations 17a and 20 [1, 19]. Fourth, our anatomic pathologists were formally blinded to histologic or molecular results, which could not introduce bias; similarly, the revision of US images was performed blinded to the other results.

## Conclusion

Diagnostic value of the main four TIRADSs (ACR, EU, K, ATA) was not improved in conjunction with the evaluation of RAS mutations for preoperative risk stratification of cytologically indeterminate thyroid nodules. Specifically, RAS testing in cytologically indeterminate nodules categorized as high suspicion for malignancy by TIRADSs could be forgone to avoid unnecessary costs. Isolated RAS positivity in sonographically non-high suspicion nodules could increase unnecessary surgery due to the relatively high suspicious call rates.

## Electronic supplementary material

Below is the link to the electronic supplementary material.

Supplementary Material 1

## Data Availability

The datasets used and/or analyzed during the current study are available from the corresponding author upon reasonable request.
