# Supplementary material for: The value of ACR, European, Korean, and ATA ultrasound risk stratification systems combined with RAS mutations for detecting thyroid carcinoma in cytologically indeterminate and suspicious for malignancy thyroid nodules
Source: Hormones (Athens). 2024 Jun 17;23(4):687–97. doi: 10.1007/s42000-024-00573-8 (PMC11519098; doi:10.1007/s42000-024-00573-8)
Supplement: Supplementary file 1 — Supplementary Material 1 [file 42000_2024_573_MOESM1_ESM.docx]

**Supplementary Table 1** Histology of the whole cohort (n: 124) according to cytology and molecular testing

|  | **TIR4** (n: 24) | | | | | | | **TIR3A** (n: 55) | | | | | | | | **TIR3B** (n: 45) | | | | | |
| --- | --- | --- | --- | --- | --- | --- | --- | --- | --- | --- | --- | --- | --- | --- | --- | --- | --- | --- | --- | --- | --- |
| **Malignant** (n: 56) | ***RAS +*** (n:10) | | | ***WILD*** (n:14) | | | ***RAS +*** (n:30) | | | | | ***WILD*** (n:25) | | | ***RAS +*** (n:18) | | | | ***WILD*** (n:27) | | |
| PTC (n: 27)   - Class - Fv - Tc - Onc | 2  4 | |  | | | 5 | | | | 6  2 | 1  1 | | | | | | | 2 | | | 1  2  1 |
| NIFTP (n: 14) | | 2 4 | | | | | | | 5 | | | | | 2 | | | 1 | | | | |
| FTC (n: 13) | 2 | |  | | | 3 | | | | | 2 | | | | | | | 4 2 | | | |
| WDT-UMP (n: 2) |  | | | | |  | | | | | 1 | | | | | | | 1 | | | |
| **Benign** (n: 68) |  | | | | | | | | | | | | | | | | | | | | |
| FA (n: 57) |  | | | | 1 | | | 13 | | | | | 13 | | | 10 | | | | 20 | |
| FND (n: 8) |  | | | | 1 | | | 4 | | | | | 2 | | | 1 | | | |  | |
| CLT (n: 3) |  | | | | |  | | | | | 3 | | | | | | |  | | | |

n, number; PTC, papillary thyroid carcinoma; NIFTP, non-invasive follicular thyroid neoplasm with papillary-like nuclear feature; FTC, follicular thyroid carcinoma; WDT-UMP, well-differentiated tumor of uncertain malignant potential; FA, follicular adenoma; FND, follicular nodular disease; CLT, chronic lymphocytic thyroiditis; Class, classic; Fv, follicular variant; Tc, tall cell; Onc, oncocytic

TIR3A, low-risk indeterminate according to the Italian Consensus for the Classification and Reporting of Thyroid Cytology [12]

TIR3B, high-risk indeterminate according to the Italian Consensus for the Classification and Reporting of Thyroid Cytology [12]

TIR4, suspicious for malignancy according to the Italian Consensus for the Classification and Reporting of Thyroid Cytology [12]

RAS positive (+) were mutated cases with isolated RAS (NRAS or HRAS or KRAS) mutation

WILD type were non-mutated cases according to our assay
